# A novel risk model to predict first-ever ischemic stroke in heart failure with reduced ejection fraction

**DOI:** 10.18632/aging.202458

**Published:** 2021-02-01

**Authors:** Xiaodong Zhou, Lingfang Yu, Weizhen Hu, Ruiyu Shi, Yinan Ji, Changzuan Zhou, Chenglong Xue, Guojia Yu, Weijian Huang, Peiren Shan

**Affiliations:** 1 Department of Cardiology, The Key Laboratory of Cardiovascular Disease of Wenzhou, The First Affiliated Hospital of Wenzhou Medical University, Wenzhou 325000, Zhejiang, P.R. China; 2 Department of Nephrology, Yuying Children’s Hospital of Wenzhou Medical University, Wenzhou 325000, Zhejiang, P.R. China; 3 Department of Cardiology, Longgang People’s Hospital, Longgang 325802, Zhejiang, P.R. China

**Keywords:** predictors, score model, heart failure with reduced ejection fraction, ischemic stroke

## Abstract

Patients with heart failure are at increased risk for ischemic stroke. We aim to develop a more accurate stroke risk prediction tools identify high-risk patients with heart failure with reduced ejection fraction (HFrEF). Patient data were extracted retrospectively from the electronic medical database between January 2009 and February 2019. Univariate and multivariate Cox regression analysis were performed to identify independent predictors, which were utilized to construct a nomogram for predicting ischemic stroke. AUROC analysis was used to compare the prognostic value between the new risk score and CHADS_2_/CHA_2_DS_2_-VASc scores. In 6087 patients with HFrEF, the risk of first-ever ischemic stroke was 5.8% events/pts-years (n=468) during 8007.2 person-years follow-up. A nomogram constructed by integrating 6 variables, including age, atrial fibrillation (AF), deep vein thrombosis (DVT), d-dimer, anticoagulant use and spontaneous echocardiographic contrast (SEC)/left ventricular thrombus (LVT), exhibited a greater area under the curve of 0.727, 0.728 and 0.714 than that by CHADS_2_ score (0.515, 0.522 and 0.540), and by CHA_2_DS_2_-VASc score (0.547, 0.553 and 0.562) for predicting first-ever ischemic stroke at hospitalization, 30-day and 6-month follow-up (all p<0.001). This novel stroke risk score performed better than existing CHADS_2_/ CHA_2_DS_2_-VASc scores and showed improvement in predicting first-ever ischemic stroke in HFrEF patients.

## INTRODUCTION

Ischemic stroke is a devastating complication in patients with heart failure (HF). [1] HF is associated with over a 2-fold increased risk of ischemic stroke than those without. [2] Notably, risk of ischemic stroke could increase 17-fold within the first 30 days after HF diagnosis. [3, 4].

Stroke risk stratification using readily available clinical variables could help identify “low-risk” and “high-risk” subgroups in HF population for more regular anticoagulant strategies for stroke prevention. [5] CHADS_2_ and CHA_2_DS_2_-VASc scores was a widely useful scoring system for stratifying ischemic stroke risks in patients with atrial fibrillation (AF). [6, 7] CHADS_2_ and CHA_2_DS_2_-VASc, as a cluster of multiple stroke risk factors, was also extensively used to predict stroke in patients with heart failure with reduced ejection fraction (HFrEF) but without AF. [8–10] A potential limitation of these approaches is selection bias, because clinical features associated with non-use of anticoagulation are likely to influence stroke risk. Prior-stroke history has been shown as the most powerful independent risk factor for improving stroke prediction. However, prognostic ability of established risk stratification scores benefited from prior-stoke history may confer low-prognostic efficiency in patients without stroke history. Additionally, all patients, who have had an ischemic stroke history are at high risk of future stroke regardless of presence of AF, and for whom anticoagulation is strongly indicated. Arguably, clinicians are particularly interested in risk stratifying patients without stroke history for ischemic stroke and how much stroke risk can be reduced in those undergoing anticoagulant management.

Therefore, this study aims to develop a nomogram to predict the probability of first-ever ischemic stroke in HFrEF, to evaluate the effectiveness of anticoagulant use in patients with HFrEF for stroke prevention, and to compare the prognostic value between the resulting scoring model and CHADS_2_/CHA_2_DS_2_-VASc scores.

## RESULTS

### Population characteristic

Clinical characteristics of the patients, stratified by the clinical outcome, are shown in Table 1. Totally, 6087 patients were enrolled in the present study. Among them, the mean age was 67.2±14.3 years, 71.0% were men, 48.7% had hypertension, 16.5% had diabetes, 21.2% had AF and 2.9% had deep vein thrombosis (DVT). LVEF at preoperative was 33.5±5.7%. Mean CHADS_2_ and CHA_2_DS_2_-VASc were 2.3±0.9 and 3.4±1.5 respectively. During the 8007.2 person-years follow up, 468 (5.8% events/pts-years) patients suffered from ischemic stroke.

**Table 1:** Characteristics of study population.

	**Overall**	**Stroke (-)**	**Stroke (+)**	**P**
	6087	5619	468	
Age	67.2±14.3	67.0±14.4	69.2±12.0	<0.001
Male	4320 (71.0%)	3979 (70.8%)	341 (72.9%)	0.368
Hypertension	2964 (48.7%)	2711 (48.2%)	253 (54.1%)	0.016
Diabetes	1485 (24.4%)	1351 (24.0%)	134 (28.6%)	0.029
ICM	2136 (35.1%)	1985 (35.3%)	151 (32.3%)	0.190
AF	1291 (21.2%)	1118 (19.9%)	173 (37.0%)	<0.001
DVT	174 (2.9%)	137 (2.4%)	137(7.9%)	<0.001
SEC/LVT	327 (5.4%)	288 (5.1%)	39 (8.3%)	0.005
CKD	1484 (24.4%)	1387 (24.7%)	97 (20.7%)	0.057
Smoking	2083 (34.2%)	1913 (34.0%)	170 (36.3%)	0.335
Drinking	1294 (21.3%)	1168 (20.8%)	126 (26.9%)	0.003
CHADS_2_	2.3±0.9	2.3±0.9	2.5±0.9	<0.001
CHA_2_DS_2_-VASc	3.4±1.5	3.3±1.5	3.5±1.4	0.023
Hemoglobin, g/L	120.5±29.5	120.2±29.8	124.5±25.5	0.001
Platelet, 10^9^/L	199.4±88.2	199.4±88.7	199.4±82.0	0.996
BNP, ng/mL	982.0 (390.0,2380.5)	986.0 (388.0,2410.0)	959.0 (390.8,1934.3)	0.299
TG, mmol/L	1.4±0.9	1.4±0.9	1.4±1.0	0.891
TC, mmol/L	4.2±1.3	4.2±1.3	4.2±1.3	0.805
LDL-C, mmol/L	2.5±1.0	2.5±1.0	2.5±0.9	0.339
CRP, mg/L	19.0 (6.6,51.4)	18.9 (6.7,52.4)	20.8 (5.7,46.7)	0.925
Cr, μmol/L	86.0 (69.0,118.0)	86.0 (69.0,118.0)	87.0 (68.0,114.0)	0.343
AST, U/L	25.0 (15.0,46.0)	25.0 (15.0,47.0)	23.0 (14.0,44.0)	0.124
Glucose, mmol/L	6.7±3.4	6.7±3.4	7.0±3.5	0.112
D-dimer	1.3 (0.7, 2.5)	1.2 (0.7, 2.5)	1.4 (0.8, 3.3)	0.003
Fibrinogen	4.3±1.5	4.3±1.5	4.4±1.5	0272
LVEF, %	33.5±5.7	33.5±5.7	33.8±5.5	0.175
Anticoagulant use	750 (12.3%)	729 (13.0%)	21 (4.5%)	<0.001
Antiplatelet use	2811 (46.2%)	2621 (46.6%)	190(40.6%)	0.012
Statins use	3150 (51.7%)	2898 (51.6%)	252 (53.8%)	0.360

Data are presented as mean±SD, median (interquartile range) or n (%). Abbreviations: ICM, ischemic cardiomyopathy; AF, atrial fibrillation; DVT, deep vein thrombosis; SEC, spontaneous echocardiographic contrast; LVT, left ventricular thrombus; CKD, chronic kidney disease; BNP, type B natriuretic peptide; TG, triglyceride; TC, total cholesterol, LDL-C, low-density lipoprotein cholesterol; CRP, C-reactive protein; Cr, creatinine; AST, aspartate aminotransferase; LVEF, left ventricular ejection fraction.

Compared with patients without ischemic stroke, those with ischemic stroke were older (69.2±12.0 vs 67.0±14.4 years, p <0.001), had a higher prevalence of hypertension (54.1% vs 48.2%; p=0.016), diabetes (28.6% vs 24.0%; p=0.029), AF (37.0% vs 19.9%; p<0.001), DVT (7.9% vs 2.4%; p<0.001) and SEC/LVT (8.3% vs 5.1%; p < 0.001), increased hemoglobin (124.5±25.5 vs 120.2±29.8g/L, p=0.001) and d-dimer levels [1.4 (0.8, 3.3) vs 1.2 (0.7, 2.5); p=0.003], but lower rate of anticoagulant use (4.5% vs 13.0%; p < 0.001) and antiplatelet use (40.6% vs 46.6%; p=0.012).

### Independent predictors for first-ever ischemic stroke in HFrEF

As shown in Table 2, 9 variables (age, hypertension, diabetes, AF, DVT, SEC/LVT, d-dimer levels, anticoagulant use, antiplatelet use) entered the Cox regression model. In univariate Cox regression analysis, age per 1 year (HR=1.018, 95% CI 1.011–1.025, p<0.001), hypertension (HR=1.189, 95% CI 0.991-1.426, p=0.063), diabetes (HR=1.275, 95% CI 1.043–1.559, p<0.001), AF (HR=2.257, 95% CI 1.870–2.723, p<0.001), DVT (HR=4.131, 95% CI 2.945-5.794, p<0.001), SEC/LVT (HR=1.806, 95% CI 1.301-2.508, p<0.001), drinking (HR=1.246, 95% CI 1.016-1.529, p=0.035), hemoglobin per 1 unit (HR=1.003, 95% CI 1.000-1.007, p=0.047), d-dimer levels per 1 unit (HR=1.060, 95% CI 1.041–1.079, p<0.001), anticoagulant use (HR=0.294, 95% CI 0.190-0.455, p<0.001) were found to be potential risk factors for stroke. In multiple Cox regression analyses, 2 continuous variables (age and d-dimer) and 4 categorical variables (AF, DVT, SEC/LVT and anticoagulant use) remained independent predictors of ischemic stroke to construct the nomogram.

**Table 2:** Univariate cox analysis of potential clinical predictors of first-ever ischemic stroke in HFrEF.

	**HR**	**95%CI**	**P value**
Age	1.018	1.011-1.025	<0.001
Hypertension	1.189	0.991-1.426	0.063
Diabetes	1.275	1.043-1.559	0.018
AF	2.257	1.870-2.723	<0.001
DVT	4.131	2.945-5.794	<0.001
SEC/LVT	1.806	1.301-2.508	<0.001
Drinking	1.246	1.016-1.529	0.035
Hemoglobin	1.003	1.000-1.007	0.047
D-dimer	1.060	1.041-1.079	<0.001
Anticoagulant use	0.294	0.190-0.455	<0.001

Abbreviations as in Table 1.

### Construction of the new scoring model

**Figure 1:**
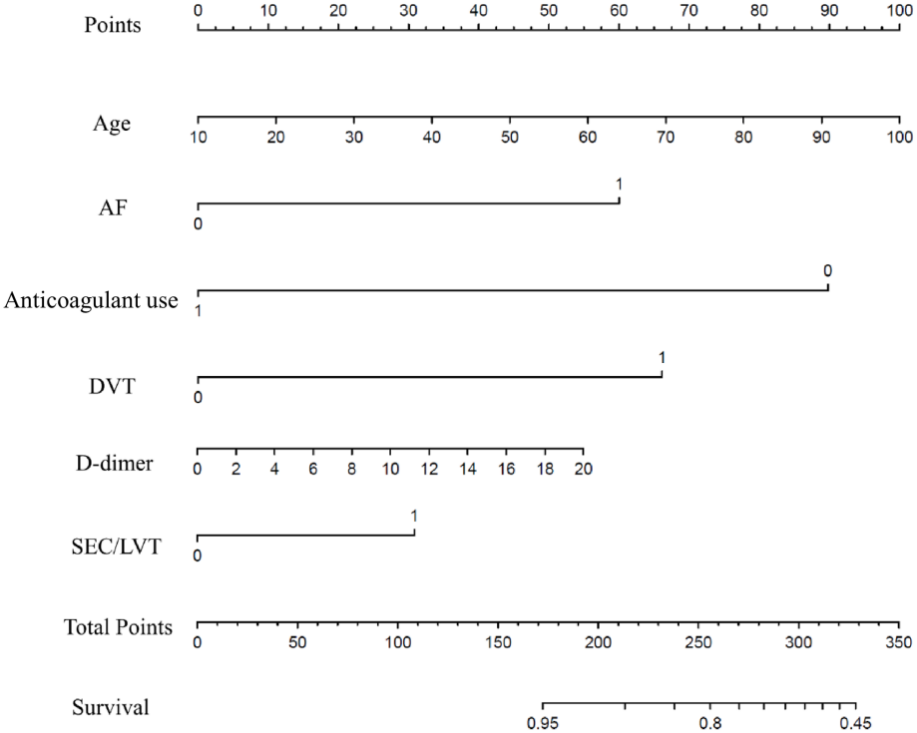
**Nomogram model for first-ever ischemic stroke prediction in HFrEF.** Abbreviations: HFrEF: heart failure with reduced ejection fraction.

According to multivariate Cox regression (Table 3), the final model included age per 1 year (HR=1.029, 95% CI 1.011–1.049, p<0.001), AF (HR=2.874, 95% CI 2.284–3.616, p<0.001), DVT (HR=3.193, 95% CI 2.010-5.075, p<0.001), SEC/LVT (HR=1.722, 95% CI 1.113–2.665, p=0.015), and d-dimer levels per 1 unit (HR=1.050, 95% CI 1.025–1.075, p<0.001) and anticoagulant use (HR=0.206, 95% CI 0.130-0.326, p<0.001) through a prognostic nomogram (Figure 1). The nomogram was created by assigning a graphic preliminary score to each of the 6 predictors with a point range from 0 to 100.

**Table 3:** Multivariate analysis for the construction of the novel stroke risk score.

	**HR**	**95%CI**	**P value**
Age per 1 year	1.029	1.011-1.049	<0.001
AF	2.874	2.284-3.616	<0.001
Anticoagulant use	0.206	0.130-0.326	<0.001
DVT	3.193	2.010-5.075	<0.001
D-diner per 1 unit	1.050	1.025-1.075	<0.001
SEC/LVT	1.722	1.113-2.665	0.015

Abbreviations as in Table 1.

### Prognosis performance of this new scoring model

**Figure 2:**
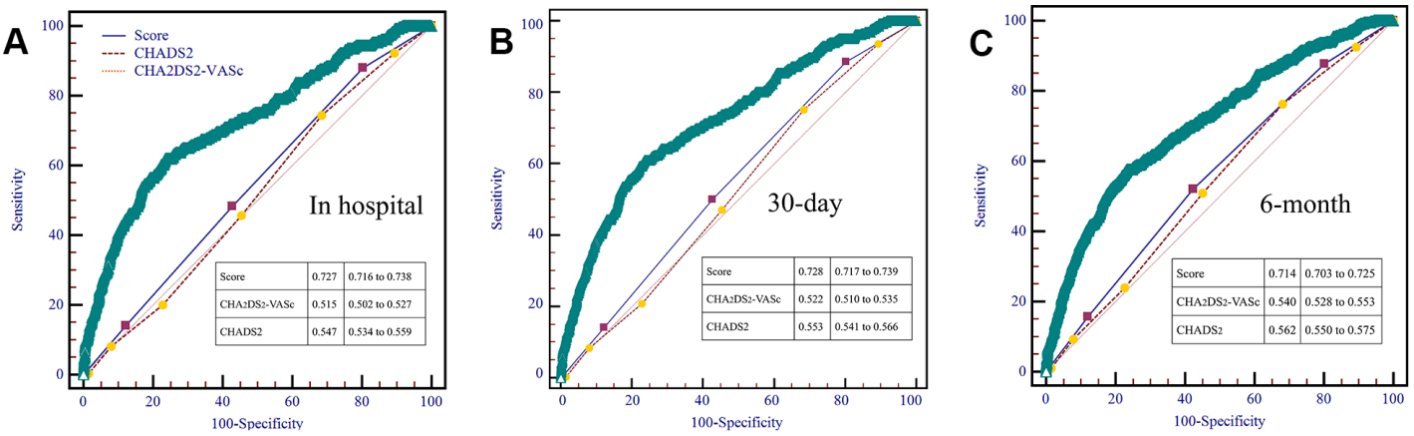
AUROC curves comparing prediction efficacy between our risk model and existing CHADS2 and CHA2DS2-VASc scores in hospital (**A**), 30-day (**B**) and 6-month (**C**) follow-up.

**Figure 3:**
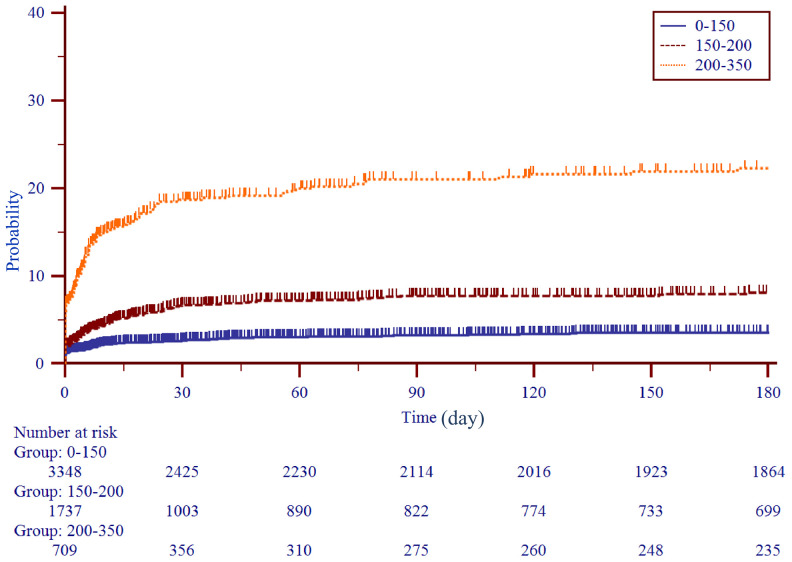
**Kaplan-Meier curves stratified by three subgroups by score levels (0-150, 150-200, 200-350).**

To use the nomogram, the first variable was located. A straight-line was then drawn upwards to the point’s axis to determine the points received for the variable. The process was repeated for the other variables and all points were then tallied to generate the total score. The sum numbers were located on the total points axis and a line was drawn downward to the survival axes and converted into an individual probability of stroke. According to the scores, subjects were divided into high, moderate and low-risk groups (0-150,150-200 and 200-350), with first-ever stroke incidence of 4.9%, 8.2% and 18.9%. Cumulative incidence curves for the entire population cohorts stratified by highest, moderate and low-risk groups are shown in Figure 2. During the first 6 months, patients with 200-350 scores had a significantly higher risk of ischemic stroke than individuals with 150-200 and 0-150 scores (P<0.001). As part of the assessment of its discriminator power, the new scoring system was compared to the CHADS_2_ and CHA_2_DS_2_-VASc score though area under the receiver operating curve (AUROC) analysis (Figure 3). AUROC of CHADS_2_ score and CHA_2_DS_2_-VASc score was 0.515 (95% CI 0.502-0.527) and 0.547 (95% CI 0.534-0.559) at hospitalization, 0.522 (95% CI 0.510-0.535) and 0.553 (95% CI 0.541-0.566) at 30-day follow-up, and 0.540 (95% CI 0.528-0.533) and 0.562 (95% CI 0.550-0.575) at 6-month follow-up. Compared with the two aforementioned scores, the new score exhibited a greater AUROC of 0.727 (95% CI 0.716-0.738), 0.728 (95% CI 0.717-0.739) and 0.714 (95% CI 0.703-0.725) for predicting first-ever ischemic stroke at hospitalization, during the 30-day and 6-month follow-up, respectively (all p<0.001).

## DISCUSSION

### Main finding

We developed a novel risk score for predicting first-ever stroke in a large cohort of HFrEF patients with or without anticoagulant use. The score included 2 continuous variables (age and d-dimer) and 4 categorical variables (AF, DVT, SEC/LVT and anticoagulant use). This scoring model predicted first-ever ischemic stroke with a significantly higher accuracy than the guideline-recommended CHADS_2_ and CHA_2_DS_2_-VASc risk model.

### Risk factors from CHADS_2_ and CHA_2_DS_2_-VASc scores

As is well-known, age independently influences stroke outcome and was presented in CHA_2_DS_2_-VASc scores as 1 score for age 65–74 and 2 scores for age ≥75 years. In contrast to CHADS_2_ and CHA_2_DS_2_-VASc scores, using continuous variable, the score of age in this novel score is dynamic with the opportunity to allow monitoring of the patient's annual changes in risk of future events. The correlation between DVT and ischemic stroke was also addressed by recent literatures. [11–13] Potential underlying mechanisms include prothrombotic tendency, inflammation activity or endothelial damage [11–14]. In this study, DVT was associated with over 3-fold increased risk and was added into the construction of our resulting scoring model. Three previously identified clinical risk factors (gender, hypertension and diabetes) from CHA_2_DS_2_-VASc risk were not included in the present evaluation, as they have not displayed independent associations with stroke in presence of other risk factors. Thus, these factors were excluded from the construction of the scoring model since they do not provide additional prognostic value in risk prediction for stroke and.

### SEC/LVT and d-dimer are two novel risk factors for stroke risk stratification in HFrEF

Patients with severe left ventricular dysfunction tend to have higher rates of SEC/LVT, which frequently was associated with higher ischemic stroke risk in patients with HFrEF. [15–18] Prognostic utility of LVT for stroke prediction also had been investigated among patients with dilated cardiomyopathy. [15] SEC is not infrequently observed with echocardiography in patients with HFrEF. Similar to LVT, SEC has also been recognized to exist in left ventricle with high incidence of thromboembolic events. [19–21] The presence of SEC/LVT may indicate fibrinogen concentration and a trigger mechanism for hypercoagulability [7, 15]. Moreover, d-dimer level also reflects increases in blood coagulation and degradation of fibrin, and thus, could be used as marker of thrombosis. [22, 23, 26] Additional studies have consistently shown that elevated d-dimer level is a determinant of the incidence of ischemic stroke not only in the general population but also in patients with HF. [23, 24].

### Quantitative evaluation of antithrombotic agents for stroke risk in HFrEF

Due to lack of findings in randomized trials, anticoagulants have not been included in international treatment recommendations for HF patients without AF. [25–28] However, the WARCEF sub-study of HF patients in sinus rhythm reported that longer time in the therapeutic range among patients allocated to warfarin reduced the risk of the ischemic stroke and also improved net clinical benefit. [29] In our scoring model, quantitative evaluation can be conducted in the context of the potential benefit of anticoagulant use to determine optimal anticoagulant therapy. This can be mirrored by one paradigmatic example. A 70-year old (68 points) patient without stroke history, diagnosed as AF (60 points), DVT (70 points), SEC/LVT (30 points), with d-dimer>20 (58 points) and without anticoagulant use (0 points) would have a total nomogram score of 218 and a following probability of stroke >10%. Conversely, a patient underwent anticoagulant would arrive at a total nomogram score of 121 and a following probability of adverse outcome approximating <5%.

### Limitation

This study has several limitations. Firstly, this study was retrospective and observational contributing to potential withdraw bias due to lost to follow-up. Secondly, this scoring model was developed from a single-center data without external validation, thereby limiting generalizability our findings. Future external validation studies should be performed in other cohorts and in patients of other ethnicities. Finally, it also remains uncertain about the influence of different dosage and duration of anticoagulant therapy for stroke prediction. High quality prospective research is needed to address unanswered questions to optimize anticoagulant therapy.

## CONCLUSIONS

In summary, we derived the scoring model for stratifying the risk of first-ever ischemic stroke in patients with HFrEF. This novel score showed an improvement of discriminative accuracy when compared to existing scores.

## MATERIALS AND METHODS

### Study design

**Figure 4:**
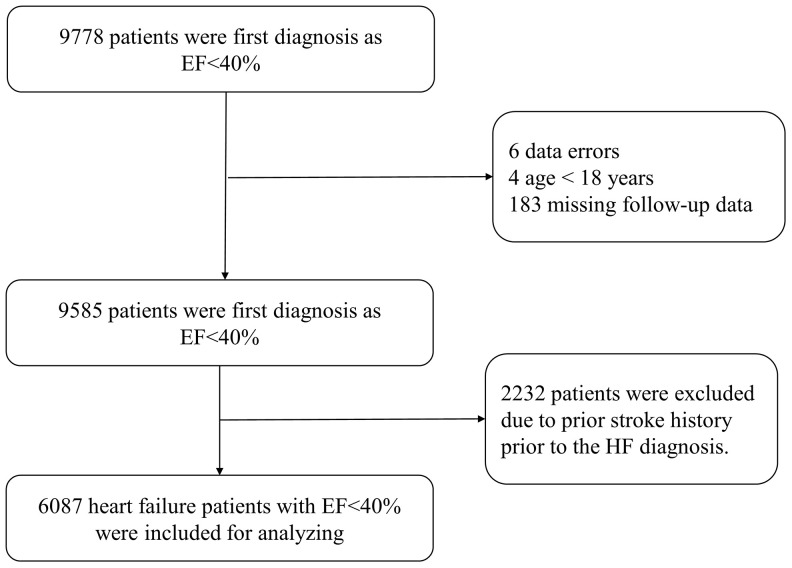
**Study flow chart.**

Data from the electronic medical records database at the First Affiliated Hospital of Wenzhou Medical University, obtained between January 2009 and February 2019, were retrospectively analyzed, which contains age, gender, previous medical history, laboratory markers, diagnosis, treatment, and follow-up data. We included all patients with a baseline mean left ventricle ejection fraction (LVEF) of <40% by quantitative echocardiography assessment. We excluded patients with age of <18 years, history of stroke prior to the HF diagnosis, presence of thrombus in left atrium or right cardiac cavity, presence of prosthetic valves, infective endocarditis or cardiac tumors, incomplete echocardiography examination and laboratory parameters, or missing relevant clinical data including follow-up data. Overall, 18097 echocardiographic records with ejection fraction (EF) <40% were extracted. Of which, 8319 reports were excluded due to repetition from the same patients. As shown in Figure 4, 9778 patients were diagnosis as EF<40% at baseline was prescreened and then were subject to exclusion criteria above, 193 patients were excluded due to data error, age < 18 years or missing follow-up data. In addition, 2232 patients were excluded history of stroke prior to the HF diagnosis. Finally, 6087 heart failure patients with EF<40% were included for analysis. For each patient, the CHADS_2_ and CHA_2_DS_2_-VASc scores were calculated. [6, 7] Based on the CHADS_2_ score, patients were given one point for congestive heart failure (CHF), hypertension, age ≥75 years, and diabetes, and two points for previous stroke or thromboembolic events (TE). Based on the CHA_2_DS_2_-VASc score, patients were given one point for CHF, hypertension, diabetes, vascular disease, age 65–74, and female gender, and two points for previous stroke or TE and age ≥75 years. This investigation conforms to the principles outlined in the Declaration of Helsinki, and was approved by the ethical committee of the First Affiliated Hospital of Wenzhou Medical University Ethical Committee.

### Definitions

The primary end point was defined as ischemic stroke at hospitalization, 30-day, 6-month and subsequently the remain period of follow-up. The nomogram was established based on outcomes at 30-day. Ischemic stroke diagnosed as clinically relevant focal neurological symptoms detected by computed tomography or magnetic resonance imaging and confirmed by a neurologist. Ischemic cardiomyopathy (ICM) was defined as CHF in the presence of cardiomyopathy associated with a documented history of myocardial infarction, coronary revascularization, or obstructive coronary artery disease (>50% stenosis) [30]. Chronic kidney disease (CKD) was defined as estimated glomerular filtration rate (eGFR) less than 60 mL/min/1.73m^2^. [31] LVEF was determined using the biplane Simpson’s method in the apical 2-chamber view. HFrEF was defined as EF with LVEF<40%. [32] Spontaneous echocardiographic contrast (SEC) was defined by dynamic smoke-like echoes with characteristic swirling motion distinct from white noise artifact. [33] LVT was diagnosed as an echogenic mass adjacent to but distinguishable from left ventricular endocardium in an area of wall-motion abnormality. Anticoagulants were used according to patients’ indications and contraindications from the current guidelines and in combination with the patients’ intentions. For high-risk patients, the use of anticoagulants would be taken under guidance of specialist physician. Some patients with atrial fibrillation (AF) or LVT were not on use of regular anticoagulant, the reasons were determined as high risk of bleeding, irregular medication, and refuse anticoagulation. Anticoagulants included warfarin and the novel oral anticoagulants (NOAC) such as dabigatran and rivaroxaban, which was available in Chinese markets.

### Statistical analysis

Baseline characteristics were described using mean±SD for continuous variables and frequency (%) for categorical variables. Student’s *t* and chi-square tests were performed to determine significant differences between groups accordingly. Non-normally distributed variables were presented as median (quartile range) and were compared using the Mann Whitney U test. The associations of clinical meaningful variables were enrolled into the univariate Cox regression models. Then the variables, which were found to be significantly associated with stroke, were entered as potential independent variables in multivariate Cox regression models. Based on the regression coefficients obtained in the multivariable regression model, the survival nomogram was generated to obtain survival probability estimations. The predictive performance of the nomogram was assessed by the area under the receiver operating curve (AUROC) and Kaplan-Meier survival analyses. A two-side p<0.05 was considered as statistically significant. All analyses were performed with SPSS software (SPSS version 23.0 for Windows), MedCale software (MedCale version 11.4 for Windows) and R software (R 3.3.1 version Development Core Team).
